# Clinical effect modifiers of antibiotic treatment in patients with chronic low back pain and Modic changes - secondary analyses of a randomised, placebo-controlled trial (the AIM study)

**DOI:** 10.1186/s12891-020-03422-y

**Published:** 2020-07-13

**Authors:** Lars Christian Haugli Bråten, Lars Grøvle, Ansgar Espeland, Are Hugo Pripp, Margreth Grotle, Christian Helllum, Anne Julsrud Haugen, Anne Froholdt, Mads Peder Rolfsen, Øystein Petter Nygaard, Olav Lutro, Per Martin Kristoffersen, Audny Anke, Elina Iordanova Schistad, Jan Sture Skouen, Jens Ivar Brox, John-Anker Zwart, Kjersti Storheim, The AIM-study group, Maja Wilhelmsen, Terese Fors, Guro Kjos, Ida Beate Østhus, Gunn Hege Marchand, Britt Elin Lurud, Fredrik Granvigen, Hege Andersen, Vidar Rao, Thomas Istvan Kadar, Siv Krüger Claussen, Erling Andersen, Nils Vetti, Jörg Aßmus, Sigrun Randen, Hilde Presberg, Monica Wigemyr, Linda Margareth Pedersen, Bendik Slagsvold Winsvold, Karianne Wiger Gammelsrud, Maria Dehli Vigeland, Benedicte Alexandra Lie, Siri Tennebø Flåm, Magnus Dehli Vigeland, Marianne Thorsø, Knut Morten Huneide, Veronica Sørensen, Thor Einar Holmgard

**Affiliations:** 1 FORMI, Oslo University Hospital HF, Ulleval, Bygg 37b, Postbox 4956, Nydalen, 0424 Oslo, Norway; 2 Faculty of Medicine, University of Oslo, PO BOX 1078 Blindern, 0316 Oslo, Norway; 3 Department of Rheumatology, Østfold Hospital Trust, PB 300, 1714 Grålum, Norway; 4 Department of Radiology, Haukeland University Hospital, Jonas Liesvei 65, 5021 Bergen, Norway; 5 Department of Clinical Medicine, University of Bergen, Klinisk institutt 1, Postboks 7804, NO-5020 Bergen, Norway; 6 Oslo Centre of Biostatistics and Epidemiology, Research Support Services, Oslo University Hospital, Postbox 4950 Nydalen, 0424 Oslo, Norway; 7 Department of Physiotherapy, Oslo Metropolitan University, PO box 4 St. Olavs plass, NO-0130 Oslo, Norway; 8 Department of Orthopaedic surgery, Oslo University Hospital, Postbox 4956, Nydalen, 0424 Oslo, Norway; 9 Department of Physical Medicine and Rehabilitation, Drammen Hospital, Vestre Viken Hospital Trust Drammen, Postboks 800, 3004 Drammen, Norway; 10 Department of Neurosurgery, St. Olavs University Hospital, Postbox 3250 Torgarden, NO-7006 Trondheim, Norway; 11 Department of Neuromedicine and Movement Science, Norwegian University of Science and Technology (NTNU), Faculty of Medicine and Health Sciences, N-7491 Trondheim, Norway; 12 National Advisory Unit on Spinal Surgery, St Olavs Hospital, Postboks 3250 Torgarden, 7006 Trondheim, Norway; 13 Stavanger University Hospital, Helse Stavanger HF, Postboks 8100, 4068 Stavanger, Norway; 14 Department of Rehabilitation, University Hospital of North Norway, Postboks 100, 9038 Tromsø, Norway; 15 Faculty of Health Sciences, Department of Clinical Medicine, UiT The Arctic University of Norway, 9037 Tromsø, Norway; 16 Department of Physical Medicine and Rehabilitation, Oslo University Hospital HF, Ulleval, Postbox 4956, Nydalen, 0424 Oslo, Norway; 17 Department of Physical Medicine and Rehabilitation, Haukeland University Hospital, Helse Bergen HF, Box 1, Bergen, Norway; 18 Department of Global Public Health and Primary Care, University of Bergen, Postboks 7804, NO-5020 Bergen, Norway

**Keywords:** Chronic low back pain, Modic changes, Antibiotic, Effect modifier, Subgroup, Randomised, Infection

## Abstract

**Background:**

Randomised trials on antibiotic treatment for patients with chronic low back pain and vertebral endplate changes visible on MRI (Modic changes) have shown mixed results. A possible explanation might be a real treatment effect in subgroups of the study populations. The purpose of the present study was to explore potential clinical effect modifiers of 3-months oral amoxicillin treatment in patients with chronic low back pain and type I or II Modic changes at the level of a previous lumbar disc herniation.

**Methods:**

We performed analyses of effect modifiers on data from AIM, a double-blind parallel-group multicentre trial. One hundred eighty patients with chronic low back pain, previous disc herniation, Modic change type I (*n* = 118) or type II (*n* = 62) were randomised to 3-months oral treatment with 750 mg amoxicillin (*n* = 89) or placebo (*n* = 91) three times daily. The primary outcome was the Roland-Morris Disability Questionnaire (RMDQ) score (possible values 0–24) at 1-year follow-up in the intention-to-treat population. The predefined minimal clinically important between-group mean difference was 4 RMDQ points (not reached in the primary analysis of AIM). Predefined baseline characteristics were analysed as potential effect modifiers, four primary (type I Modic changes, previous disc surgery, positive pain provocation test, high CRP) and five exploratory (disturbed sleep, constant low back pain, short duration of low back pain, younger age, and male) using ANCOVA with interaction terms.

**Results:**

None of the four primary potential effect modifiers had strong evidence of modifying the treatment effect. In patients younger than 40 years the difference in mean RMDQ score between the treatment groups was − 4.0 (95%CI, − 6.9 to − 1.2), compared to − 0.5 (95%CI, − 2.3 to 1.3) in patients 40 years or older, both in favour of amoxicillin treatment (exploratory analysis).

**Conclusions:**

We did not find evidence for convincing clinical effect modifiers of antibiotic treatment in patients with chronic low back pain and Modic changes. Our results for younger age in these explorative analyses should not affect clinical treatment decisions without confirmation in future studies.

**Trial registration:**

ClinicalTrials.gov NCT02323412, First registered 23 December 2014.

## Background

In most patients with chronic low back pain no specific or underlying disease can be found. An efficient management is often difficult and current treatment options for low back pain offer at best only small to moderate reductions in pain and disability [1].

Accordingly, there are ongoing attempts to identify subgroups with specific clinical characteristics that could lead to effective treatments. A suggested subgroup is patients with vertebral bone marrow changes extending from the endplate (Modic changes) visible on magnetic resonance imaging (MRI), which may be associated with low back pain [2]. A prominent hypothesis for the etiology of Modic changes is a low-grade bacterial infection of the adjacent intervertebral disc [3]. A disc herniation is a suggested precondition for the bacteria to enter into the disc.

A randomised placebo controlled trial published in 2013 reported that three months of antibiotic treatment offered substantial improvement of symptoms in patients with chronic low back pain and type I Modic changes [4]. The AIM-study (Antibiotics In Modic changes), a reassessment and replication study of the former trial from 2013, did not find any clinically important difference in outcome between the treatment groups at three or 12 months follow-up [5]. There was however a small difference between the groups and an increased variance in outcome measure in the amoxicillin group compared to the placebo group, that may suggest a possible treatment effect in subgroups [6]. There is also a biological rationale for the heterogeneity of treatment effect, as even positive biopsy studies in patients with Modic changes mostly have a large proportion of negative samples [7]. There have not been any reported attempts so far to find effect modifiers of antibiotic treatment in low back pain based on randomised trials.

The present study was hypothesis-setting [8], meaning that we attempted to find candidate effect modifiers to be verified in further hypothesis-testing studies. We sought subgroups with treatment response, not diagnostic subgroups. The objective of this study was to explore potential clinical effect modifiers of three months oral amoxicillin treatment in patients with chronic low back pain and type I or II Modic changes at the level of a previous lumbar disc herniation included in the AIM-study.

## Methods

Our hypothesis was that one or more of nine predefined clinical characteristics (described below) modified the efficacy of three months oral amoxicillin treatment in patients with chronic low back pain and type I or II Modic changes at the level of a previous lumbar disc herniation.

### Study design and setting

The present hypothesis-setting analyses were based on data from the AIM-study, a multicentre, randomised, double-blind, placebo-controlled, parallel-group trial with a treatment phase (three months) and a follow-up phase (nine months). Trial methods have been detailed previously [9]. The AIM-study was performed in accordance with the Helsinki Declaration. This included being registered at ClinicalTrials.gov in December 2014 under the identifier: NCT02323412, approved by the Regional Committees for Medical Research Ethics - South East Norway (2014/158/REK sør-øst) before being commenced and that all participants gave written informed consent. This study is reported in accordance with the CONSORT guidelines [10] and recommendations for research method framework for studies of subgroups in low back pain [8].

### Study population

Patients were recruited from outpatient clinics at six hospitals in Norway from June 2015 to September 2017. We included patients with age 18 to 65 years, low back pain for > 6 months, and low back pain intensity ≥5 on a 0–10 Numerical Rating Scale, a lumbar disc herniation on MRI in the former two years, and type I and/or type II Modic changes (with height ≥ 10% of vertebral height and diameter > 5 mm) at the herniated disc level. Patients were excluded if they had surgery for disc herniation in the last year, or any specific diagnosis that could explain patient’s low back symptoms (i.e. tumor, fracture, spondyloarthritis, infection, spinal stenosis) [5].

### Randomisation, masking and procedures

Patients were allocated to either three months oral treatment with amoxicillin 750 mg three times daily or placebo. Allocation was concealed by a computer generated allocation sequence number and stratified by Modic change type (I or II) and previous disc herniation surgery (yes or no). All care providers, research staff, statisticians, and patients were blinded to group allocation during the data collection and analysis. Both treatment groups received identical capsules, containers, and labels.

### Outcomes and data collection

The score on the Norwegian version of the Roland Morris Disability Questionnaire (RMDQ) at 1-year follow-up was used as primary outcome. Secondary outcomes were Oswestry Disability Index (ODI) 2.0 and low back pain intensity (0–10 NRS). Serum concentrations of CRP were measured with the same method for all study centres (Roche Diagnostics) detecting levels down to 0.6 mg/L. We defined type I Modic changes as primary (most extensive) or secondary type I Modic changes. Patients with type II Modic changes, but not type I Modic changes, were defined as belonging to the type II Modic change group. Two experienced radiologists independently evaluated Modic changes and trial eligibility based on a standardized baseline 1.5 T MRI, and discussed and solved all disagreements on eligibility. There was no change in the trial measurements after trial start.

### Potential effect modifiers

We preselected, predefined, pre-categorized and ordered four baseline characteristics as primary potential effect modifiers and five as explorative potential effect modifiers, based on a biological rationale and previous literature (Table 1). An additional three variables (NSAIDs consumption during treatment phase, compliance and treatment effect at 3 months) described in the Statistical Analysis Plan were not analysed, as they were measured after baseline [18].

**Table 1:** Predefined potential effect modifiers of interest

**Potential effect modifier**	**Biological rationale**
**Primary analyses**	
1. Modic changes type I	
We expected a larger treatment effect in type I compared to type II.	Type I Modic changes are more strongly associated with low back pain [11]. In animal models, injecting *C.acne* into intervertebral discs induced type I Modic changes [12, 13].
2. Previous disc surgery at level with Modic changes	
We expected a larger treatment effect in patients with previous disc surgery compared to those without.	Low-grade discitis might be a complication of disc surgery due to introduction of bacteria into the disc during the surgical procedure. The randomised trial from 2013 with a high number of patients with previous surgery found effect of antibiotic treatment [4], while a case series with few patients with previous surgery was negative [14].
3. Positive pain provocation test	
We expected a larger treatment effect in patients with a positive Springing test (patient reported pain with pressure applied to lumbar transverse processes) compared to those with a negative Springing test.	Spinal tenderness may indicate regular spondylodiscitis [15]. Springing test is found to be borderline significant discriminator between patients with and without Modic changes [16].
4. Elevated CRP (C-reactive protein)	
We expected a larger treatment effect in those with higher CRP. The predefined cut-off values for CRP were changed from 3 mg/L and 10 mg/L to 5 mg/L due to too few patients in the predefined categories.	CRP in serum is associated with bacterial infection and inflammation.
**Exploratory analyses**	
1. Disturbed sleep	
We expected a larger treatment effect in those with disturbed sleep than in those without. Disturbed sleep was defined as a ≥ 2 score on the Oswestry Disability Index item 7 (sleep scale), i.e. less than 6 h sleep to no sleep because of pain.	Night-time pain may indicate infectious spondylodiscitis [15].
2. Constant low back pain	
We expected a larger treatment effect in those patients with constant low back pain compared to those with fluctuating low back pain.	Constant pain may indicate regular spondylodiscitis [15].
3. Short duration of low back pain	
We expected a larger treatment effect in those with short duration of symptoms compared to those with longer duration of symptoms. The predefined categorization (< 1 year, 1–2 years and ≥ 2 years) was dichotomized into < 2 years and ≥ 2 years due to too few patients with symptoms < 1 year.	Recent disc herniation could have increased perfusion in the disc as part of disc repair, thereby increasing absorption of amoxicillin.
4. Younger age	
We expected a larger treatment effect in patients < 40 years of age compared to those ≥40 years of age.	*C.acne* could be more prevalent in discs of young patients [17].
5. Male gender	
We expected a larger treatment effect in men compared to women.	*C.acne* could be more prevalent in discs in men than in women [17].

### Statistical analysis

We pre-specified all statistical analyses in a Statistical Analysis Plan in advance of database locking [19]. We used the same 50 imputed sets as for the original trial, where missing RMDQ values (13/180) and missing values in the secondary outcomes were imputed with a multiple imputation model using predictive mean matching as described in the Statistical Analysis Plan for the main results [20]. Missing values of the potential effect modifiers were not imputed. All analyses were performed and all figures were made using software package Stata version 15.

The effect of each of the nine potential effect modifiers was analysed with an ANCOVA model on the intention-to-treat population using RMDQ scores at 1-year follow up as the dependent variable. Independent variables included the baseline RMDQ score, treatment group, the effect modifier, treatment group multiplied with the effect modifier (interaction term), and the stratification variables embedded in the randomisation (Modic change type and previous disc surgery). The hypothesis of whether there is a difference in the treatment effect across levels of the potential effect modifier was assessed by the interaction term [18, 21]. We also report mean differences between the antibiotic and the placebo group for patients in each category of the subgroup by pairwise comparison of margins in the model with the interaction term (stratified analysis). Using the analysis of Modic change type as an example, the stratified analysis compared the predicted mean in the amoxicillin group with the predicted mean in the placebo group for only those patients who had type I Modic changes (and then separately for those who had type II Modic changes). The stratified analyses intend to answer “what is the effect of the treatment in each category of the subgroup?” The significance test of the interaction term intends to answer “was the observed difference in the treatment effect between the two categories of the subgroup due to a statistical significant effect modification?” Our analyses should not be confused with analyses of prognostic factors, which is another type of subgroup analyses in which it is not possible to elucidate if the observed subgroups are due to the treatment or not [8].

To explore whether any effect modifier was consistent across related outcomes we repeated the analyses using ODI and low back pain intensity as dependent variables [18]. In addition, to assess if any effect modification remained when adjusted for the other potential effect modifiers (independency of effect) [18], we performed ANCOVA with baseline RMDQ score, treatment groups, all nine effect modifiers, all nine potential effect modifier-treatment group interactions terms, and the stratification variables as independent variables. We ignored any findings in effect modifiers that did not have a clinically relevant result in the main analysis, as we intended to just evaluate consistency and independency of the results (of individual effect modifiers for RMDQ) and wanted to reduce the problem with multiple testing. We regarded a real effect of antibiotics more likely if present across many outcome measures.

As in the original trial, we predefined the minimal clinically important between-group difference in mean RMDQ score as 4. The AIM-study was powered for secondary analyses separately for type I and type II Modic changes, in which 66 patients were needed to detect (β = 0.1, α = 0.05) a difference of 4 (standard deviation 5) in mean RMDQ score between the two treatment groups. To equally power effect modifier analyses with interaction terms at the identical effect of the modifier as for the between-group difference, a roughly four times larger sample (66 × 4 = 264 patients) would be needed [22]. However, since the present sample (*n* = 180) give a somewhat lower power of about 75% to assess effect modifier analyses (assuming evenly sized groups and an identical effect size of the modifier as for the between-group difference), we focused on the size and direction of the effects, rather than relying only on the statistical significance of the interaction term. The statistical power was likely somewhat enhanced as we included baseline values of the outcome, and the stratification variables, as covariates. We also analysed the potential effect modifiers in a prioritized order. As the analyses were hypothesis-setting only, we did not adjust the significance level due to multiple testing [8].

## Results

Of 180 included patients, 89 were randomised to amoxicillin and 91 to placebo. One patient in the amoxicillin group and one patient in the placebo group did not report the baseline RMDQ. Four patients in the amoxicillin group and seven patients in the placebo group did not report RMDQ at 1-year follow up. All randomised patients were included in the intention-to-treat analyses (multiple imputations were used to account for missing values in the primary and secondary outcomes).

The distribution of the potential effect modifiers at baseline is presented in Table 2. Elevated CRP was present in 14/89 (17%) in the amoxicillin group and in 7/91 (8%) in the placebo group. The distribution of baseline characteristics within each potential effect modifier is presented in Table S1 in the Supplementary Appendix.

**Table 2:** Distribution of the potential effect modifiers according to treatment groups

	**Amoxicillin (*n*** **= 89)** **Number of patients**	**Placebo (*n*** **= 91)** **Number of patients**
Primary effect modifiers		
Modic changes type I		
Yes	58	60
No	31	31
Missing	0	0
Previous disc surgery		
Yes	18	20
No	71	71
Missing	0	0
Pain provocation test		
Negative	15	10
Positive	74	81
Missing	0	0
CRP		
< 5 mg/L	70	82
≥ 5 mg/L	14	7
Missing	5	2
Exploratory effect modifiers		
Disturbed sleep		
No	42	47
Yes	46	42
Missing	1	2
Pain characteristics		
Fluctuating	70	63
Constant	19	26
Missing	0	2
Duration of low back pain		
< 2 years	31	25
≥ 2 years	58	65
Missing	0	1
Age		
< 40 years	28	27
≥ 40 years	61	64
Missing	0	0
Gender		
Male	36	39
Female	53	52
Missing	0	0

*CRP* C-reactive protein

*ODI* Oswestry Disability Index. Score from 0 to 100. Higher scores indicate more severe pain and disability

### Primary analysis

**Fig. 1:**
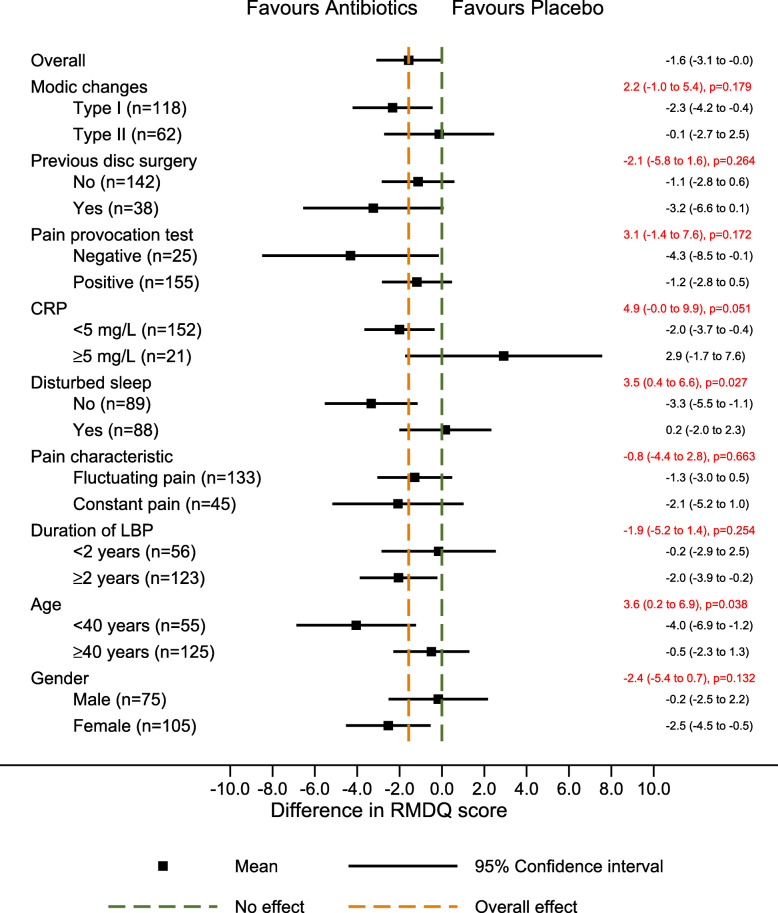
Forest plot with results for RMDQ (primary outcome). The difference in mean RMDQ score between the treatment groups (size of treatment effect) with 95% confidence interval is shown in black on the right for each of the two categories of each potential effect modifier. The difference in size of treatment effect between the two categories (estimated by the interaction term), with 95% confidence interval and p-values is shown in red. RMDQ Roland-Morris Disability Questionnaire. Score from 0 to 24. Higher scores indicate more severe pain and disability. CRP C-reactive protein. ODI Oswestry Disability Index. Score from 0 to 100. Higher scores indicate more severe pain and disability. LBP Low back pain

The treatment effect (the adjusted mean difference in RMDQ score between the amoxicillin group and the placebo group at 1 year) for patients with type 1 Modic changes was − 2.3 (95% CI, − 4.2 to − 0.4), with type 2 Modic changes − 0.1 (95% CI, − 2.7 to 2.5), and with a difference between the two of 2.2 (95% CI, − 1.0 to 5.4) (estimates from the interaction term) (Fig. 1). The similar treatment effect for patients with previous disc surgery was − 3.2 (95% CI, − 6.6 to 0.1), and for patients without previous disc surgery − 1.1 (95% CI, − 2.8 to 0.6; interaction term, − 2.1; 95% CI, − 5.8 to 1.6), for patients with negative pain provocation test − 4.3 (95% CI, − 8.5 to − 0.1), for patients with positive pain provocation test − 1.2 (95% CI, − 2.8 to 0.5; interaction term, 3.1; 95% CI, − 1.4 to 7.6), for patients with low CRP − 2.0 (95% CI, − 3.7 to − 0.4), and for patients with elevated CRP 2.9 (95% CI, − 1.7 to 7.6; interaction term, 4.9; 95% CI, 0.0 to 9.9). The direction of effect for pain provocation test and CRP was opposite of the predefined hypothesis.

### Exploratory analysis

The adjusted mean difference in RMDQ score between the treatment groups for those with disturbed sleep was 0.2 (95% CI, − 2.0 to 2.3), and for those with undisturbed sleep − 3.3 (95% CI, − 5.5 to − 1.1; interaction term, 3.5; 95% CI, 0.4 to 6.6) (opposite direction of the predefined hypothesis) (Fig. 1). In younger patients, i.e. < 40 years, the adjusted mean difference was − 4.0 (95% CI, − 6.9 to − 1.2), and in those ≥40 years old − 0.5 (95% CI, − 2.3 to 1.3; interaction term, 3.6; 95% CI, 0.2 to 6.9), in favour of amoxicillin (same direction of effect as hypothesised).

In the multivariate analysis including all effect modifiers and their interaction terms the adjusted mean difference in RMDQ score between the treatment groups was 3.5 (95% CI, − 0.1 to 7.0) points larger for those younger than 40 years compared with those aged 40 years and older (Table S2). The relationship between age and change in RMDQ from baseline to one year is shown in Fig. S1 in the Supplementary Appendix.

**Fig. 2:**
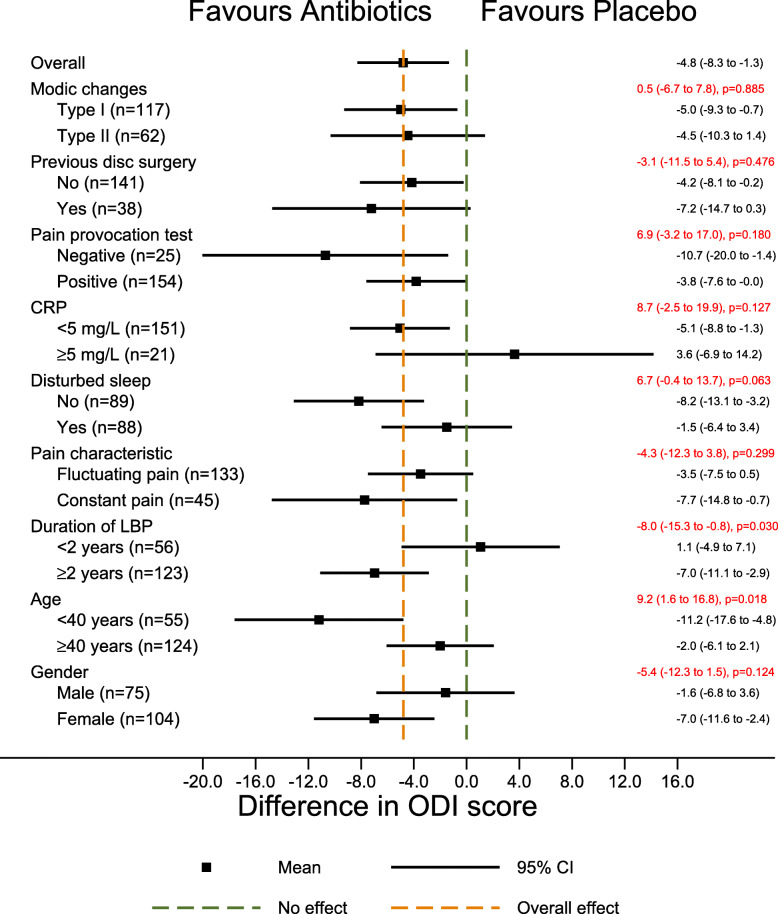
Forest plot with results for ODI (secondary outcomes). The difference in mean ODI score between the treatment groups (size of treatment effect) with 95% confidence interval is shown in black on the right for each of the two categories of each potential effect modifier. The difference in size of treatment effect between the two categories (estimated by the interaction term), with 95% confidence interval and *p*-values is shown in red. CRP C-reactive protein. ODI Oswestry Disability Index. Score from 0 to 100. Higher scores indicate more severe pain and disability. LBP Low back pain

**Fig. 3:**
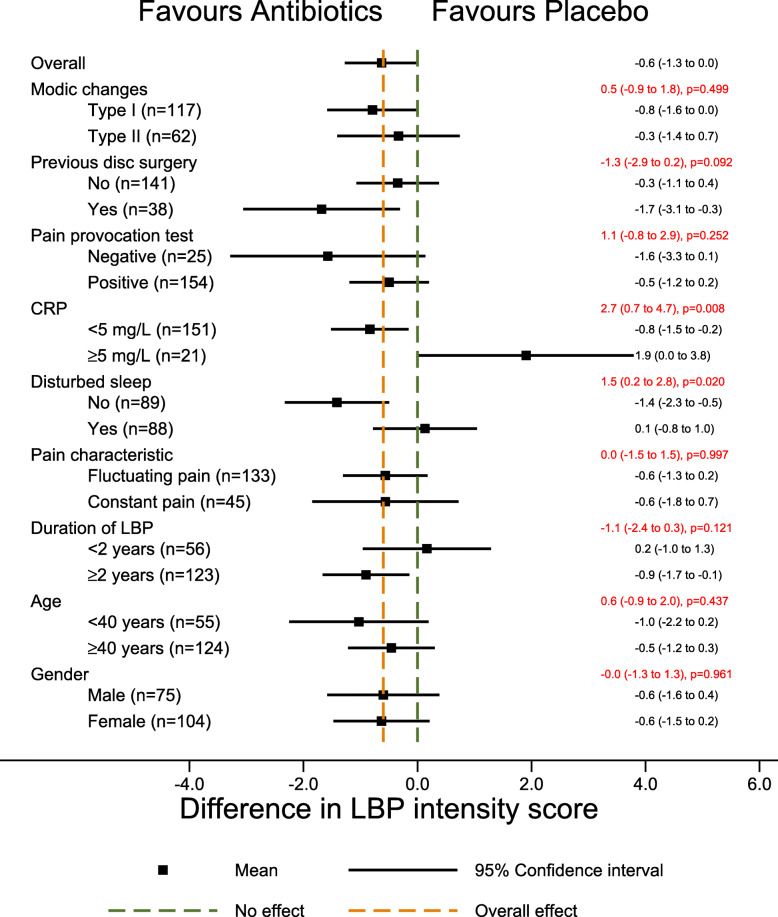
Forest plot with results for low back pain intensity (secondary outcome). The difference in mean low back pain intensity score between the treatment groups (size of treatment effect) with 95% confidence interval is shown in black on the right for each of the two categories of each potential effect modifier. The difference in size of treatment effect between the two categories (estimated by the interaction term), with 95% confidence interval and p-values is shown in red. CRP C-reactive protein. ODI Oswestry Disability Index. Score from 0 to 100. Higher scores indicate more severe pain and disability. LBP Low back pain

The primary potential effect modifiers had no or only a small effect on ODI or low back pain intensity in the hypothesized direction. The treatment effect did not change a great deal across age categories (9.2; 95%CI 1.6 to 16.8) with a moderate treatment effect in younger patients (− 11.2; 95%CI − 17.6 to 4.8) on ODI (Fig. 2), but not on low back pain intensity (Fig. 3).

## Discussion

In this study, none of the four primary potential effect modifiers (type I Modic changes, previous disc surgery, positive pain provocation test, high CRP) had a clinically relevant or statistically significant impact on the effect of antibiotic treatment in patients with chronic low back pain and type I or II Modic changes at the level of a previous lumbar disc herniation. Patients with low CRP had better treatment effect than those with high CRP, opposite of our prespecified direction, and mainly driven by better RMDQ scores in the placebo compared to the amoxicillin group for those with high CRP, which we find biologically implausible. In the exploratory analyses we observed a small effect modification of disturbed sleep in the opposite of the prespecified direction. We observed a modifying effect of age; patients younger than 40 years had a clinically relevant and statistically significant effect of antibiotic treatment on the RMDQ and ODI, but not on low back pain intensity, and the effect on RMDQ diminished when all effect modifiers were analysed simultaneously. A treatment effect in younger patients might seem biologically plausible, since they might be more likely to have *C.acnes* in their discs (Table 1) and relatively less likely to have Modic changes as part of age related degeneration compared to older patients [17, 23]. However, these explorative results should be interpreted with great caution, and require confirmation in future studies. Further, our predefined subgroups should be interpreted as surrogate subgroups, meaning that there is no claim of a causal mechanism involved [24]. E.g there is no claim that age in itself cause effect modification, but rather that age may be associated with an unknown factor that could cause effect modification.

The most important limitation of this study is the lack of a confirmed bacterial infection in the disc prior to antibiotic treatment. Second, there is a further limitation due to possibly insufficient statistical power, leading to increased risks of type I and II errors [25]. Our results indicating age as an effect modifier may represent a type I error due to multiple testing. The present findings should therefore not influence clinical practice. They could still be important in the design and interpretation of future trials on antibiotic treatment for patients with low back pain and Modic changes, and of studies on the etiology of Modic changes. Third, we cannot exclude a possible treatment effect in subgroups were the lower end of the confidence interval crosses the predefined limit for clinically important difference (e.g. type 1 Modic changes, previous disc surgery and constant pain). However, the most likely treatment effects in these subgroups are still below clinical importance.

Some of the inclusion- and exclusion criteria used in the AIM study limit the generalizability of the results of the present study. AIM only included patients with a previous disc herniation within the last two years. This could theoretically make the trial more likely to detect a treatment effect of antibiotics, since the infection theory of Modic changes suggests that a disc herniation is needed for bacteria to enter into the disc [3, 26]. However, this theory has not been confirmed and it is uncertain how our results for treatment modifiers would have been in patients without a previous disc herniation. Further, our results are not generalizable for patients not fulfilling the inclusion criteria, ie lower baseline low back pain intensity, outside our age limits, and with other comorbidities.

Microbiological studies on disc biopsies show mixed results, and it is still uncertain whether the observed *C.acnes* in such studies are partly or completely explained by contamination. However, proteomic analyses of degenerated discs did find evidence of response to gram positive bacterial infection that is difficult to explain by contamination [27]. Further trials with antibiotic treatment in back pain should preferably be performed on patients with confirmed infection, either microbiologically or other methods.

## Conclusions

We did not find evidence for convincing clinical effect modifiers of antibiotic treatment in this secondary analysis of the AIM study. Age may possibly act as an effect modifier, requiring attention in further studies, but the present results should not affect clinical practice without further confirmation.

## Supplementary information

**Additional file 1: Table S1.** Baseline characteristics in the antibiotic and placebo group, for each potential effect modifier. **Table S2.** Multivariate model of primary outcome (RMDQ). **Figure S1.** Change in RMDQ score (from baseline to 1-year) by age.

## Data Availability

Requests to access data should be addressed to kjersti.storheim@medisin.uio.no. De-identified individual participant data (including data dictionary) will be available to medical researchers by request in accordance with local registration and ethical approval, when the article has been published until 1st of July, 2029. All proposals requesting data access will need to specify an analysis plan and will need approval of the scientific board before any data can be released.

## Abbreviations

AIM: Antibiotics in Modic changes
MRI: Magnetic Resonance Image
RMDQ: Roland-Morris Disability Questionnaire
CRP: C-reactive protein
ANCOVA: Analysis of covariance
REK: Regionale komiteer for medisinsk og helsefaglig forskningsetikk (Regional Committees for Medical and Health research Ethics)
CONSORT: Consolidated Standards of Reporting Trials
ODI: Oswestry Disability Index
NSAIDs: Non-steroidal anti-inflammatory drugs
C.acnes: Cutibacterium acnes
