# Supplementary material for: Clinical effect modifiers of antibiotic treatment in patients with chronic low back pain and Modic changes - secondary analyses of a randomised, placebo-controlled trial (the AIM study)
Source: BMC Musculoskelet Disord. 2020 Jul 13;21:458. doi: 10.1186/s12891-020-03422-y (PMC7359501; doi:10.1186/s12891-020-03422-y)
Supplement: Supplementary file 1 — Additional file 1: Table S1. Baseline characteristics in the antibiotic and placebo group, for each potential effect modifier. Table S2. Multivariate model of primary outcome (RMDQ). Figure S1. Change in RMDQ score (from baseline to 1-year) by age. [file 12891_2020_3422_MOESM1_ESM.docx]

**Supplementary Appendix**

## **Results:**

Table S1 - Baseline characteristics in the antibiotic and placebo group, for each potential effect modifier

| Baseline characteristic |  | Type I MCs | Previous disc surgery | Pain provoca-tion test positive | CRP ≥5 mg/L | Disturbed sleep | Constant LBP | Short duration of LBP | Younger age | Male gender |
| --- | --- | --- | --- | --- | --- | --- | --- | --- | --- | --- |
| Age | Amoxicillin | 45 (9.3), n=58 | 47 (9.7), n=18 | 45 (9.1), n=74 | 44 (13), n=14 | 46 (9.4), n=46 | 44 (9.9), n=19 | 44 (9.2), n=31 | 35 (4.1), n=28 | 46 (9), n=36 |
|  | Placebo | 46 (9.3), n=60 | 44 (9.8), n=20 | 45 (9), n=81 | 45 (4.9), n=7 | 47 (8.3), n=42 | 46 (9.8), n=26 | 46 (9.3), n=25 | 34 (3.8), n=27 | 45 (8.6), n=39 |
| Women | Amoxicillin | 35/58 (60%) | 11/18 (61%) | 44/74 (59%) | 10/14 (71%) | 28/46 (61%) | 12/19 (63%) | 21/31 (68%) | 18/28 (64%) | 0/36 (0%) |
|  | Placebo | 35/60 (58%) | 11/20 (55%) | 49/81 (60%) | 5/7 (71%) | 25/42 (60%) | 15/26 (58%) | 18/25 (72%) | 15/27 (56%) | 0/39 (0%) |
| Body Mass Index | Amoxicillin | 26 (4.2), n=58 | 26 (3), n=18 | 26 (4.1), n=74 | 28 (5.2), n=14 | 27 (3.9), n=46 | 26 (3.4), n=19 | 26 (5.2), n=31 | 26 (4.4), n=28 | 27 (3.8), n=36 |
|  | Placebo | 25 (3.7), n=60 | 25 (3.5), n=20 | 26 (4.2), n=80 | 28 (3.5), n=7 | 27 (4.4), n=42 | 24 (3.3), n=26 | 26 (4.1), n=24 | 24 (2.5), n=27 | 27 (4.3), n=38 |
| Smoking, yes | Amoxicillin | 18/58 (31%) | 2/18 (11%) | 22/74 (30%) | 6/14 (43%) | 13/46 (28%) | 7/19 (37%) | 7/31 (23%) | 6/28 (21%) | 8/36 (22%) |
|  | Placebo | 13/60 (22%) | 7/20 (35%) | 18/81 (23%) | 1/7 (14%) | 13/42 (32%) | 9/26 (36%) | 2/25 (8%) | 7/27 (26%) | 9/39 (24%) |
| RMDQ score (0-24) | Amoxicillin | 12.9 (4.3), n=58 | 13.0 (5.5), n=18 | 13.4 (4.4), n=74 | 13.5 (4.5), n=14 | 13.8 (4.8), n=46 | 14.2 (3.7), n=19 | 13.4 (3.6), n=30 | 12.9 (4.7), n=28 | 12.0 (4.7), n=36 |
|  | Placebo | 12.3 (3.7), n=60 | 13.4 (4.2), n=19 | 12.8 (3.7), n=80 | 13.9 (4.6), n=7 | 13.8 (4.1), n=42 | 12.8 (4.0), n=26 | 12.9 (4.5), n=24 | 13.0 (3.5), n=26 | 12.8 (3.7), n=39 |
| ODI score (0-100) | Amoxicillin | 31 (11), n=58 | 32 (10), n=18 | 33 (11), n=74 | 37 (11), n=14 | 37 (12), n=46 | 37 (12), n=19 | 31 (9.2), n=30 | 31 (11), n=28 | 30 (12), n=36 |
|  | Placebo | 30 (9.9), n=58 | 33 (11), n=20 | 32 (10), n=79 | 34 (8.3), n=7 | 36 (10), n=42 | 35 (8.5), n=25 | 32 (12), n=25 | 30 (9.4), n=27 | 31 (11), n=39 |
| Pain intensity (0-10) | Amoxicillin | 6.5 (1.1), n=58 | 6.2 (1.2), n=18 | 6.6 (1), n=74 | 6.6 (1.1), n=14 | 6.5 (1.3), n=46 | 7.1 (.86), n=19 | 6.4 (1.1), n=31 | 6.2 (1.2), n=28 | 6.3 (1.3), n=35 |
|  | Placebo | 6.3 (1.3), n=59 | 6.2 (1.4), n=20 | 6.4 (1.5), n=80 | 6 (1.3), n=7 | 6.7 (1.4), n=42 | 6.7 (1.5), n=26 | 6.1 (1.5), n=25 | 6.4 (1.7), n=27 | 6.3 (1.6), n=39 |
| Educational level |  |  |  |  |  |  |  |  |  |  |
| Primary school (9 years) | Amoxicillin | 8/57 (14%) | 2/18 (11%) | 9/73 (12%) | 3/14 (21%) | 4/45 (8.9%) | 3/19 (16%) | 3/30 (10%) | 4/28 (14%) | 5/36 (14%) |
|  | Placebo | 4/58 (6.9%) | 3/20 (15%) | 8/79 (10%) | 0/7 (0%) | 4/41 (9.8%) | 6/25 (24%) | 1/25 (4%) | 2/27 (7.4%) | 4/38 (11%) |
| High school (12 years) | Amoxicillin | 21/57 (37%) | 7/18 (39%) | 31/73 (42%) | 7/14 (50%) | 19/45 (42%) | 7/19 (37%) | 16/30 (53%) | 12/28 (43%) | 13/36 (36%) |
|  | Placebo | 27/58 (47%) | 10/20 (50%) | 38/79 (48%) | 4/7 (57%) | 19/41 (46%) | 9/25 (36%) | 11/25 (44%) | 14/27 (52%) | 24/38 (63%) |
| College or university (<4 years) | Amoxicillin | 17/57 (30%) | 7/18 (39%) | 24/73 (33%) | 3/14 (21%) | 14/45 (31%) | 5/19 (26%) | 8/30 (27%) | 7/28 (25%) | 12/36 (33%) |
|  | Placebo | 11/58 (19%) | 4/20 (20%) | 14/79 (18%) | 2/7 (29%) | 9/41 (22%) | 6/25 (24%) | 4/25 (16%) | 5/27 (19%) | 5/38 (13%) |
| University (≥4 years) | Amoxicillin | 11/57 (19%) | 2/18 (11%) | 9/73 (12%) | 1/14 (7.1%) | 8/45 (18%) | 4/19 (21%) | 3/30 (10%) | 5/28 (18%) | 6/36 (17%) |
|  | Placebo | 16/58 (28%) | 3/20 (15%) | 19/79 (24%) | 1/7 (14%) | 9/41 (22%) | 4/25 (16%) | 9/25 (36%) | 6/27 (22%) | 5/38 (13%) |
| Comorbidity* |  |  |  |  |  |  |  |  |  |  |
| Score 1 (back pain only) | Amoxicillin | 40/58 (69%) | 15/18 (83%) | 48/74 (65%) | 8/14 (57%) | 28/46 (61%) | 14/19 (74%) | 22/31 (71%) | 22/28 (79%) | 26/36 (72%) |
|  | Placebo | 40/60 (67%) | 13/20 (65%) | 52/81 (64%) | 3/7 (43%) | 25/42 (60%) | 19/26 (73%) | 15/25 (60%) | 20/27 (74%) | 25/39 (64%) |
| Score 2 | Amoxicillin | 13/58 (22%) | 3/18 (17%) | 20/74 (27%) | 3/14 (21%) | 14/46 (30%) | 4/19 (21%) | 7/31 (23%) | 4/28 (14%) | 7/36 (19%) |
|  | Placebo | 17/60 (28%) | 6/20 (30%) | 25/81 (31%) | 4/7 (57%) | 14/42 (33%) | 6/26 (23%) | 9/25 (36%) | 5/27 (19%) | 11/39 (28%) |
| Score >2 | Amoxicillin | 5/58 (8.6%) | 0/18 (0%) | 6/74 (8.1%) | 3/14 (21%) | 4/46 (8.7%) | 1/19 (5.3%) | 2/31 (6.5%) | 2/28 (7.1%) | 3/36 (8.3%) |
|  | Placebo | 3/60 (5%) | 1/20 (5%) | 4/81 (4.9%) | 0/7 (0%) | 3/42 (7.1%) | 1/26 (3.8%) | 1/25 (4%) | 2/27 (7.4%) | 3/39 (7.7%) |
| Former disc surgery | Amoxicillin | 10/58 (17%) | 18/18 (100%) | 16/74 (22%) | 2/14 (14%) | 14/46 (30%) | 3/19 (16%) | 7/31 (23%) | 3/28 (11%) | 7/36 (19%) |
|  | Placebo | 12/60 (20%) | 20/20 (100%) | 17/81 (21%) | 1/7 (14%) | 8/42 (19%) | 8/26 (31%) | 3/25 (12%) | 8/27 (30%) | 9/39 (23%) |
| Emotional distress(HSCL-25 ≥1.75) § | Amoxicillin | 14/58 (25%) | 7/18 (39%) | 22/74 (30%) | 6/14 (43%) | 18/46 (39%) | 8/19 (42%) | 9/31 (29%) | 9/28 (32%) | 3/36 (8.3%) |
|  | Placebo | 12/60 (20%) | 5/20 (25%) | 19/81 (23%) | 1/7 (14%) | 14/42 (33%) | 8/26 (31%) | 7/25 (28%) | 9/27 (33%) | 13/39 (33%) |
| FABQ physical activity (0-24) ¶ | Amoxicillin | 12 (5.9), n=58 | 10 (5.9), n=18 | 12 (5.7), n=74 | 12 (6.2), n=14 | 12 (5.8), n=46 | 13 (4.6), n=19 | 9.6 (4.3), n=31 | 12 (6.4), n=28 | 12 (6.1), n=36 |
|  | Placebo | 13 (6), n=59 | 14 (5.7), n=20 | 13 (5.9), n=80 | 15 (7), n=7 | 13 (5.6), n=42 | 13 (6.5), n=26 | 12 (5.9), n=25 | 14 (4.8), n=27 | 13 (5.1), n=39 |
| FABQ work(0-24) ¶ | Amoxicillin | 17 (11), n=57 | 21 (11), n=18 | 19 (12), n=72 | 22 (13), n=14 | 20 (11), n=44 | 22 (11), n=19 | 17 (11), n=30 | 17 (12), n=28 | 15 (11), n=35 |
|  | Placebo | 18 (12), n=59 | 22 (13), n=20 | 19 (12), n=79 | 15 (11), n=7 | 19 (12), n=41 | 22 (13), n=25 | 18 (12), n=25 | 21 (10), n=27 | 22 (12), n=39 |
| Duration of back pain in years | Amoxicillin | 2.8(1.5-5.2) | 2.9(1.5-4.8) | 2.6(1.3-6) | 2.5(1.3-6) | 4(2.2-7) | 5.7(3-12) | 1.2(.83-1.5) | 2.4(1.1-3.7) | 3(1.6-6.1) |
|  | Placebo | 4(2-8) | 4.3(2.4-5) | 3.8(1.7-7.3) | 2.5(.92-3) | 3.5(1.3-7) | 5(3-9.5) | 1.3(1-1.5) | 4(2-5) | 5(2.5-10) |
| Physical work load |  |  |  |  |  |  |  |  |  |  |
| Mostly sitting | Amoxicillin | 28/51 (55%) | 8/16 (50%) | 29/62 (47%) | 5/11 (45%) | 18/37 (49%) | 5/15 (33%) | 13/27 (48%) | 12/25 (48%) | 18/32 (56%) |
|  | Placebo | 18/49 (37%) | 3/15 (20%) | 24/68 (35%) | 2/6 (33%) | 13/34 (38%) | 7/22 (32%) | 10/23 (43%) | 4/22 (18%) | 9/28 (32%) |
| Job requires a lot of walking | Amoxicillin | 12/51 (24%) | 4/16 (25%) | 17/62 (27%) | 2/11 (18%) | 9/37 (24%) | 6/15 (40%) | 8/27 (30%) | 9/25 (36%) | 4/32 (13%) |
|  | Placebo | 14/49 (29%) | 5/15 (33%) | 17/68 (25%) | 1/6 (17%) | 10/34 (29%) | 6/22 (27%) | 7/23 (30%) | 4/22 (18%) | 6/28 (21%) |
| Job requires a lot of walking and lifting | Amoxicillin | 9/51 (18%) | 2/16 (13%) | 14/62 (23%) | 4/11 (36%) | 8/37 (22%) | 3/15 (20%) | 5/27 (19%) | 4/25 (16%) | 7/32 (22%) |
|  | Placebo | 13/49 (27%) | 6/15 (40%) | 23/68 (34%) | 3/6 (50%) | 11/34 (32%) | 7/22 (32%) | 6/23 (26%) | 11/22 (50%) | 10/28 (36%) |
| Job requires physically heavy work | Amoxicillin | 2/51 (3.9%) | 2/16 (13%) | 2/62 (3.2%) | 0/11 (0%) | 2/37 (5.4%) | 1/15 (6.7%) | 1/27 (3.7%) | 0/25 (0%) | 3/32 (9.4%) |
|  | Placebo | 4/49 (8.2%) | 1/15 (6.7%) | 4/68 (5.9%) | 0/6 (0%) | 0/34 (0%) | 2/22 (9.1%) | 0/23 (0%) | 3/22 (14%) | 3/28 (11%) |
| Modic type I group | Amoxicillin | 58/58 (100%) | 10/18 (56%) | 50/74 (68%) | 7/14 (50%) | 29/46 (63%) | 15/19 (79%) | 18/31 (58%) | 21/28 (75%) | 23/36 (64%) |
|  | Placebo | 60/60 (100%) | 12/20 (60%) | 53/81 (65%) | 4/7 (57%) | 24/42 (57%) | 18/26 (69%) | 14/25 (56%) | 17/27 (63%) | 25/39 (64%) |
| Presence of leg pain | Amoxicillin | 41/58 (71%) | 16/18 (89%) | 58/74 (78%) | 12/14 (86%) | 39/46 (85%) | 14/19 (74%) | 21/31 (68%) | 19/28 (68%) | 27/36 (75%) |
|  | Placebo | 43/60 (73%) | 17/20 (85%) | 62/81 (78%) | 4/7 (57%) | 35/42 (83%) | 21/26 (81%) | 17/25 (68%) | 21/27 (78%) | 33/39 (85%) |
| Employment status |  |  |  |  |  |  |  |  |  |  |
| Working full time | Amoxicillin | 33/58 (57%) | 7/18 (39%) | 33/74 (45%) | 3/14 (21%) | 18/46 (39%) | 8/19 (42%) | 15/31 (48%) | 16/28 (57%) | 21/36 (58%) |
|  | Placebo | 32/60 (53%) | 6/20 (30%) | 39/81 (48%) | 1/7 (14%) | 17/42 (40%) | 12/26 (46%) | 15/25 (60%) | 15/27 (56%) | 16/39 (41%) |
| Partial sick leave | Amoxicillin | 7/58 (12%) | 5/18 (28%) | 13/74 (18%) | 2/14 (14%) | 8/46 (17%) | 2/19 (11%) | 7/31 (23%) | 3/28 (11%) | 4/36 (11%) |
|  | Placebo | 11/60 (18%) | 3/20 (15%) | 19/81 (23%) | 3/7 (43%) | 12/42 (29%) | 7/26 (27%) | 5/25 (20%) | 4/27 (15%) | 9/39 (23%) |
| Complete sick leave | Amoxicillin | 4/58 (6.9%) | 1/18 (5.6%) | 7/74 (9.5%) | 3/14 (21%) | 7/46 (15%) | 2/19 (11%) | 3/31 (9.7%) | 2/28 (7.1%) | 4/36 (11%) |
|  | Placebo | 3/60 (5%) | 2/20 (10%) | 7/81 (8.6%) | 1/7 (14%) | 4/42 (9.5%) | 2/26 (7.7%) | 4/25 (16%) | 1/27 (3.7%) | 3/39 (7.7%) |
| Disability pension | Amoxicillin | 13/58 (22%) | 3/18 (17%) | 17/74 (23%) | 5/14 (36%) | 11/46 (24%) | 5/19 (26%) | 4/31 (13%) | 5/28 (18%) | 6/36 (17%) |
|  | Placebo | 10/60 (17%) | 8/20 (40%) | 12/81 (15%) | 1/7 (14%) | 7/42 (17%) | 5/26 (19%) | 1/25 (4%) | 5/27 (19%) | 10/39 (26%) |
| Unemployed | Amoxicillin | 1/58 (1.7%) | 1/18 (5.6%) | 2/74 (2.7%) | 1/14 (7.1%) | 0/46 (0%) | 1/19 (5.3%) | 1/31 (3.2%) | 1/28 (3.6%) | 1/36 (2.8%) |
|  | Placebo | 2/60 (3.3%) | 1/20 (5%) | 3/81 (3.7%) | 1/7 (14%) | 1/42 (2.4%) | 0/26 (0%) | 0/25 (0%) | 1/27 (3.7%) | 0/39 (0%) |
| Other | Amoxicillin | 0/58 (0%) | 1/18 (5.6%) | 2/74 (2.7%) | 0/14 (0%) | 2/46 (4.3%) | 1/19 (5.3%) | 1/31 (3.2%) | 1/28 (3.6%) | 0/36 (0%) |
|  | Placebo | 2/60 (3.3%) | 0/20 (0%) | 1/81 (1.2%) | 0/7 (0%) | 1/42 (2.4%) | 0/26 (0%) | 0/25 (0%) | 1/27 (3.7%) | 1/39 (2.6%) |

Data are mean (SD), median (IQR), or no./N(%).

RMDQ=Rolland-Morris Disability Questionnaire. ODI=Oswestry Disability Index.

* Functional Comorbidity Index – Score increased by 1 for each of 18 diagnoses associated with decreased physical function.^1^

§ Emotional distress (Hopkins Symptom Checklist–25), values ≥1.75 associated with psychiatric diagnosis.^2^

¶ Fear-Avoidance Beliefs Questionnaire, higher values indicating more fear-avoidance beliefs.^3^

Table S2- Multivariate model of primary outcome (RMDQ)

| **Potential effect modifier** |  | **Model part** |  | **N** |  | **Estimates** |  | **95% Confidence**  **interval** |  | **P-value** |
| --- | --- | --- | --- | --- | --- | --- | --- | --- | --- | --- |
| Modic changes type I | | Interaction term | |  | | -2.3 | | -5.6 to 0.9 | 0.16 | |
| Yes | | Marginals | | 118 | | −2.3 | | −4.1 to −0.4 | 0.02 | |
| No | | Marginals | | 62 | | 0.1 | | −2.6 to 2.7 | 0.96 | |
|  | |  | |  | |  | |  |  | |
| Previous disc surgery | | Interaction term | |  | | -1.9 | | -5.7 to 1.9 | 0.33 | |
| Yes | | Marginals | | 142 | | −1.1 | | −2.8 to 0.6 | 0.22 | |
| No | | Marginals | | 38 | | −2.9 | | −6.3 to 0.5 | 0.09 | |
|  | |  | |  | |  | |  |  | |
| Pain provocation test | | Interaction term | |  | | 2.4 | | -2.3 to 7.0 | 0.31 | |
| Negative | | Marginals | | 25 | | −3.6 | | −7.8 to 0.7 | 0.10 | |
| Positive | | Marginals | | 155 | | −1.2 | | −2.8 to 0.5 | 0.17 | |
|  | |  | |  | |  | |  |  | |
| CRP | | Interaction term | |  | | 3.6 | | -1.2 to 8.5 | 0.14 | |
| <5 mg/L | | Marginals | | 152 | | −1.9 | | −3.6 to −0.3 | 0.02 | |
| ≥5 mg/L | | Marginals | | 21 | | 1.7 | | −2.9 to 6.3 | 0.46 | |
|  | |  | |  | |  | |  |  | |
| Disturbed sleep | | Interaction term | |  | | 2.2 | | -1.3 to 5.6 | 0.22 | |
| No | | Marginals | | 89 | | −2.6 | | −4.9 to −0.2 | 0.03 | |
| Yes | | Marginals | | 88 | | −0.4 | | −2.7 to 1.9 | 0.72 | |
|  | |  | |  | |  | |  |  | |
| Pain characteristic | | Interaction term | |  | | -0.2 | | -3.9 to 3.5 | 0.92 | |
| Fluctuating | | Marginals | | 133 | | −1.4 | | −3.2 to 0.3 | 0.11 | |
| Constant | | Marginals | | 45 | | −1.6 | | −4.8 to 1.5 | 0.31 | |
|  | |  | |  | |  | |  |  | |
| Duration of low back pain | | Interaction term | |  | | -4.1 | | -7.8 to -0.4 | 0.03 | |
| <2 years | | Marginals | | 56 | | 1.4 | | −1.5 to 4.3 | 0.35 | |
| ≥2 years | | Marginals | | 123 | | −2.8 | | −4.7 to −0.8 | 0.006 | |
|  | |  | |  | |  | |  |  | |
| Age | | Interaction term | |  | | 3.5 | | -0.1 to 7.0 | 0.055 | |
| <40 years | | Marginals | | 55 | | −3.9 | | −6.9 to −0.9 | 0.01 | |
| ≥40 years | | Marginals | | 125 | | −0.4 | | −2.2 to 1.4 | 0.64 | |
|  | |  | |  | |  | |  |  | |
| Gender | | Interaction term | |  | | -3.4 | | -6.6 to -0.3 | 0.03 | |
| Male | | Marginals | | 75 | | 0.5 | | −1.8 to 2.8 | 0.68 | |
| Female | | Marginals | | 105 | | −2.9 | | −5.0 to −0.9 | 0.005 | |

All numbers are based on the same multivariate model, where all subgroups with their interaction terms were analysed. Marginals show the estimated pairwise comparison of the predicted marginal mean in the amoxicillin vs the placebo groups, marginalised across all other interaction terms and covariates.

CRP C-reactive protein

ODI Oswestry Disability Index. Score from 0 to 100. Higher scores indicate more severe pain and disability.

LBP Low back pain

Figure S1- Change in RMDQ score (from baseline to 1-year) by age

Negative values means improvement in RMDQ (primary outcome) score from baseline to 1-year follow up. Figure shows that for patients younger than 40 years old, there was better improvement in RMDQ score in the amoxicillin group compared to the placebo group.

| RMDQ | Roland-Morris Disability Questionnaire. Score from 0 to 24. Higher scores indicate more severe pain and disability. |
| --- | --- |
| Lowess | Locally weighted scatterplot smoothing. Obtains a smoothed curve by a weighted least squares regression. |

References:

1. Groll DL, To T, Bombardier C, Wright JG. The development of a comorbidity index with physical function as the outcome. Journal of Clinical Epidemiology 2005;58:595-602.

2. Sandanger I, Moum T, Ingebrigtsen G, Dalgard OS, Sorensen T, Bruusgaard D. Concordance between symptom screening and diagnostic procedure: the Hopkins Symptom Checklist-25 and the Composite International Diagnostic Interview I. Soc Psychiatry Psychiatr Epidemiol 1998;33:345-54.

3. Waddell G, Newton M, Henderson I, Somerville D, Main CJ. A Fear-Avoidance Beliefs Questionnaire (FABQ) and the role of fear-avoidance beliefs in chronic low back pain and disability. Pain 1993;52:157-68.
